# Burden of Disease of Duchenne Muscular Dystrophy in Denmark – A National Register-Based Study of Individuals with Duchenne Muscular Dystrophy and their Closest Relatives

**DOI:** 10.3233/JND-230133

**Published:** 2024-03-05

**Authors:** Jan Håkon Rudolfsen, John Vissing, Ulla Werlauff, Charlotte Olesen, Niels Illum, Jens Olsen, Peter Bo Poulsen, Mette Strand, Alfred Peter Born

**Affiliations:** a EY, Copenhagen, Denmark; b Copenhagen Neuromuscular Center, Copenhagen University Hospital, Copenhagen, Denmark; c The Danish Rehabilitation Centre for Neuromuscular Diseases, Aarhus, Denmark; d Center for Rare Diseases and Neuropediatric Department, Aarhus University Hospital, Aarhus, Denmark; e H. C. Andersen Children’s Hospital, Odense University Hospital, Odense, Denmark; f Health & Value, Pfizer Denmark, Ballerup, Denmark; g Medical Affairs, Pfizer Denmark, Ballerup, Denmark; h Department of Child- and Adolescent Medicine, Copenhagen University Hospital, Copenhagen, Denmark

**Keywords:** Muscular dystrophy, duchenne, incidence, prevalence, cost of illness, siblings, education, parents, productivity, cost, registers, population

## Abstract

**Background:**

Duchenne Muscular Dystrophy (DMD) is a progressive genetic disease with a prevalence of 1 per 3,600–6,000 male births. Individuals with DMD are typically diagnosed at age 4–7 years; median survival is 30 years. They require multidisciplinary care, personal assistance, and often special education.

**Objective:**

The aim was to assess the burden of disease in DMD in Denmark. This includes incidence, prevalence, use of healthcare services, labour market participation, educational outcomes, and overall attributable costs due to DMD. Impact on the closest relatives (siblings and parents) was also investigated.

**Methods:**

The comprehensive Danish national health and administrative registers were used to assess the burden of disease following individuals with DMD and closest relatives from five years before, and up to 20 years after DMD diagnosis. Individuals with DMD (and relatives) from 1994–2021 were included. All outcomes were compared to matched control groups without the disease drawn from the Danish population.

**Results:**

213 unique individuals with DMD were identified. They had lower grades in school, required more special education and more healthcare and home care compared to their control group. The extra costs of special education summed to EUR 180,900 over the course of 11 years elementary school. They had an annual average productivity loss of EUR 20,200 between the age of 18 to 30. The extra healthcare costs of DMD in the 20 years after diagnosis were estimated to EUR 1,524,000. If an individual with DMD lives to be 30, total extra costs sum to EUR 2,365,800.

**Conclusions:**

Using national register data this study presented detailed results on the burden of disease of DMD, including impact on closest relatives. With 60 additional hospital admissions and 200 extra outpatient contacts in 20 years healthcare costs, but also costs of home care and special education, increases as disease progresses.

## INTRODUCTION

Duchenne Muscular Dystrophy (DMD) is a rare, progressive, genetic disease with a prevalence of 1 per 3,600–6,000 male births [1, 2]. DMD is caused by pathogenic variants in the dystrophin gene (*DMD*). This results in a complete loss of dystrophin and dystrophin-associated glycoprotein complex, which are important proteins for the structural integrity of the sarcolemma membrane. DMD is X-linked, and therefore, the disease primarily affects boys, while female carriers are unaffected or usually only mildly affected.

Early symptoms manifest as muscle weakness, difficulties to raise from floor, run and climb stairs. Cognition and language development are often affected as well [3]. The typical individuals with DMD are diagnosed at age 4–7 years and will become permanent wheelchair users in their early teens, and often use respiratory aids by their mid to late teens [4]. The disease also causes cardiomyopathy and often osteoporosis, the consequences of which is amplified by immobility and steroid treatment. Additionally, many individuals have behaviour- and learning difficulties. As a result, individuals with DMD have reduced life expectancy with a median survival age of about 30 years [5–8] and impaired quality of life and an existential worry about life [9].

Improvements in standard of care have improved the life expectancy for individuals with DMD, but there is no curative treatment [4]. Best practice treatment according to international guidelines consists of supportive treatment and managing symptoms, including the use of corticosteroid [7]. Muscle weakness and cardiopulmonary and bone-related comorbidities cause the most significant burden of illness. However, individuals with DMD require multidisciplinary patient-centred care –e.g., medical specialists, nurses, physiotherapists, speech therapists, respiratory care staff, home care assistance and dieticians, all specialised in DMD to prevent and manage symptoms [10].

The aim of this study was to assess the burden of disease in DMD. Previous studies have been conducted using survey-based approaches, while a recent review concluded that register studies are necessary to improve evidence of disease development over time [11]. Similarly, knowledge about the impact of DMD on close relatives or informal care givers as DMD progresses is sparse [12]. The present study used comprehensive, national Danish health and administrative registers to address this data source request and knowledge gap. Individuals with DMD and their close relatives were followed from five years before, and up to 20 years after DMD diagnosis. Burden of illness was measured through use and costs of primary and hospital care, prescription drugs, home care services, labour market participation and educational achievements. In addition to individuals with DMD, outcomes for parents and siblings were included as well. Furthermore, all outcomes were compared to matched control groups from the general Danish population.

## MATERIALS AND METHODS

### Study population and data sources

Individuals with DMD were identified in the Danish National Patient Register (NPR) using ICD-10 diagnosis codes [13]. Denmark has a unique ICD-10 code specifically for DMD –G71.0H. Individuals were included in the study, if they were observed on at least two occasions with this DMD diagnosis in a hospital setting. The first observation of muscular dystrophy was set as the index date if the general G71.0 predated the two observations with G71.0H. As the DMD-specific ICD-10 code was established in 1994, the study period was set from 1994 to 2021. Some individuals were first observed with a DMD diagnosis code when they were 18 years or older. This is to be expected early in the observation period, as prevalent DMD individuals are included. In reality, however, individuals with DMD are diagnosed at a much earlier age, and a diagnosis at > 18 year is likely a misdiagnosis, or inaccurate diagnosis of individuals with DMD-like conditions, such as Becker muscular dystrophy.

To retain prevalent DMD individuals early in the observation period, we included individuals who were diagnosed at > 18 years old before the year 2000 in the study population. From the year 2000 onwards, we excluded individuals diagnosed while being > 18 years old.

Parents of individuals with DMD were identified through the Danish Central Person Register (CPR). All Danish residents have a unique 10-digit personal identification number. If the parent of an individual with DMD was a Danish resident, the parent could be identified in the CPR. If a parent had more than one child with DMD, the parent was included once in the analysis, and diagnosis of the first child with DMD was used as index date.

Siblings of individuals with DMD were similarly identified through the CPR. Once the parents of individuals with DMD were identified, all children of these parents could also be identified. In the case of multiple siblings in one family, only the sibling closest in age to the individual with DMD was included in analysis.

Data on hospital contacts (inpatient and outpatient contacts) were obtained in the NPR and in addition to the NPR and CPR, data on primary care services were obtained from the Danish National Health Service Register. Data on consumption of prescription medication was obtained from the Register of Medi-cinal Product Statistics. Use of home care services was obtained from Statistics Denmark’s databases on municipality services. Home care services are registered to the individual personal identification number. Home care services are provided as either personal care services or practical assistance (cleaning, household tasks, etc.). Data on income was collected from the Income Register. Labour market participation was collected from the longitudinal database of The Danish Agency for Labour Market and Recruitment (DREAM) database. The DREAM database contains all labour market transfer payments on a weekly basis, going back to 1991 [14]. Educational performance was collected from the Education Register, Register of Primary School Grades, and Register of Special Education. Date of death was obtained from the Cause of Death Register, while emigration was observed in the Migration register.

All the above-mentioned registers contain individual-level observations noted with the personal identification number used to merge registers [15]. As the register data is routinely used in reporting and financing healthcare, education or social transfers, and all Danes are eligible to receive these services, the data is of high completeness, and cover a wide range of potential outcomes.

### Control population

For each individual with DMD, parent, and sibling, 10 controls were identified. The pool of controls consisted of the entire Danish population, who was not observed with a muscular dystrophy diagnosis. Matching variables for individuals with DMD and siblings were age, sex, region of residence at the time of diagnosis, and parents’ education. Parents were matched on age, sex, region of residence and employment status at time of diagnosis, highest obtained education, and number of children.

Exact matching was applied. For 20 individuals (individuals with DMD/parents/sibling), it was not possible to identify 10 controls. This was solved by weighting of the control observations, such that all the controls for a particular indivi-dual with DMD/parent/sibling had the same influence on the analysis, independent of the number of controls identified for each individual. For example, if 10 controls were identified for an individual with DMD/parent/sibling, each control received a weight of 1/10. If only eight controls were identified, the controls received a weight of 1/8.

### Outcomes

Incidence and prevalence of DMD were presented. Risk of death relative to the respective control groups was analysed. Furthermore, incidence of the most common comorbidities and of most frequent surgeries were presented, stratified by age group. Comorbidities were defined by ICD-10 codes, sur-geries were defined according to procedure codes. Additionally, frequent selected comorbidities and procedures for individuals with DMD was described, this included incidence of scoliosis, Achilles tendon surgery, respiratory illnesses and heart disease (see Supplementary Table 1). Hospital contacts re-gistering dependency on wheelchair or mechanical ventilator was also presented, defined through ICD-10 codes Z99.1 and Z99.3, respectively.

Inpatient hospital care was defined as hospital contacts lasting 12 hours or longer. Outpatient hospital contacts were defined as in-person contacts at a hospital or outpatient clinic. For inpatient and outpatient hospital contacts, the frequency of contacts was counted, and the costs of contacts were calculated based on Diagnosis-Related-Group (DRG) tariffs.

The costs of primary care were calculated as the reimbursement price of services provided. Costs of prescription drugs were calculated as the market price of the drug at the pharmacy, including 25% VAT.

Home care services were defined as the number of hours of home care delivered within a period. Home care is divided into two categories; a) care intended to assist in personal care, b) care intended to assist in usual activities. The number of hours was counted, and the costs of home care were calculated based on the estimated hourly costs of delivering home care.

Annual income was defined as the mean income for each individual, excluding government transfers.

To determine the extra costs related to DMD, the difference between the study groups and the matched control group was presented –i.e., the extra costs or use attributable to DMD. As DMD is a progressive disease, we conducted an age-based subgroup analysis on individuals with DMD aged 0–7 (infancy/childhood), 8–11 (childhood), 12–17 (late childhood/adolescent) and 18 plus years (young adult/adult), representing disease stages related to the international standard of care program [18–20].

Early retirement was defined as the first week when a person received disability pension. Disability pension is only granted after a physician has determined that a person has permanently reduced ability to work. Long-term sick leave was defined as sick leave lasting longer than four weeks. Unemployment was defined as persons who are in the labour force who did not engage in income-generating activities.

All costs were adjusted to 2021 level according to the consumer price index calculated by Statistics Denmark [16]. Conversion to Euro was done by 1 EUR = DKK 7.5.

Highest obtained education was a descriptive measure gathered from the Education Register. For individuals with DMD and their siblings, education performance was measured as grades obtained in elementary and high school, as well as the probability of completing selected educational thresholds such as elementary school, high school, or higher education. Incidence of special education was also described.

### Proxy outcomes

Costs of special education and costs associated with in-home mechanical respirator consist primarily of labour costs of teachers, assistants, nurses, and other personnel designated to the specific tasks. These costs were not registered in the Danish registers; however, they were expected to constitute a notable part of the total extra costs of DMD. Therefore, for the costs of special education, we calculated time spent in special education per individual included in the study and multiplied it by the average cost of special education in Denmark [17].

Similarly, with costs of respiratory treatment at home, we identified start of in-home mechanical respiratory treatment in the Danish registers and included the mean annual costs of respiratory care at home based on previous studies. Start of respiratory treatment was defined as hospital contact with procedure code BGFC3 (non-invasive ventilation). Start of in-home respirator treatment was defined as contact with one out of three highly specialised respiratory centres in Denmark in combination with a diagnosis of chronic respiratory failure, indicating start of full-time in-home mechanical respirator treatment. The costs of respirator treatment were derived from a public health technology assessment of in-home mechanical respirators in Denmark [18]. One year with a mask respirator was estimated to be EUR 125,400 (2021 price level), while one year of in-home mechanical respirator was estimated to be EUR 211,000 (2021 price level).

### Statistical analysis

Participants were followed up to five years before occurrence of the DMD diagnosis (earliest 1 January 1994) and potentially 20 years after diagnosis, unless death, emigration or end of follow-up occurred first (latest 31 December 2021). Note that data on costs were only available for the years 1994–2020.

For risk of death and early retirement, Kaplan-Meier plots were made, with hazard ratios estimated by Cox proportional hazard regression. Date of diagnosis was used as the index date for these analyses. For costs of care and use of healthcare services as well as long-term sick leave and unemployment, an ordinary least square regression model was used to estimate the mean values in each year before and after the index date. The effect attributable to DMD was calculated as the difference between DMD-related individuals and their controls. In analysis of siblings, the effect of some siblings also having DMD was adjusted for to get DMD-free sibling specific effects. Significance was determined by comparing 95% confidence intervals (CI) of the means in the respective groups.

Due to data privacy regulations, some results will be reported as ‘fewer than five cases’ or ‘< 5’. This is done to ensure anonymity of the included individuals. All analyses and data handling were done in R, version 4.2.1 (www.r-project.org).

### Ethical approval and consent

According to Danish law, approval from the Danish Data Protection Agency as well as ethical committee approval is not required for registry-based studies in Denmark. Neither is consent from individuals with DMD required as data in the national registers (pseudo-anonymized) are available for research to be used by researchers from academia and other institutions with a research access to the national registers (license).

## RESULTS

**Fig. 1:**
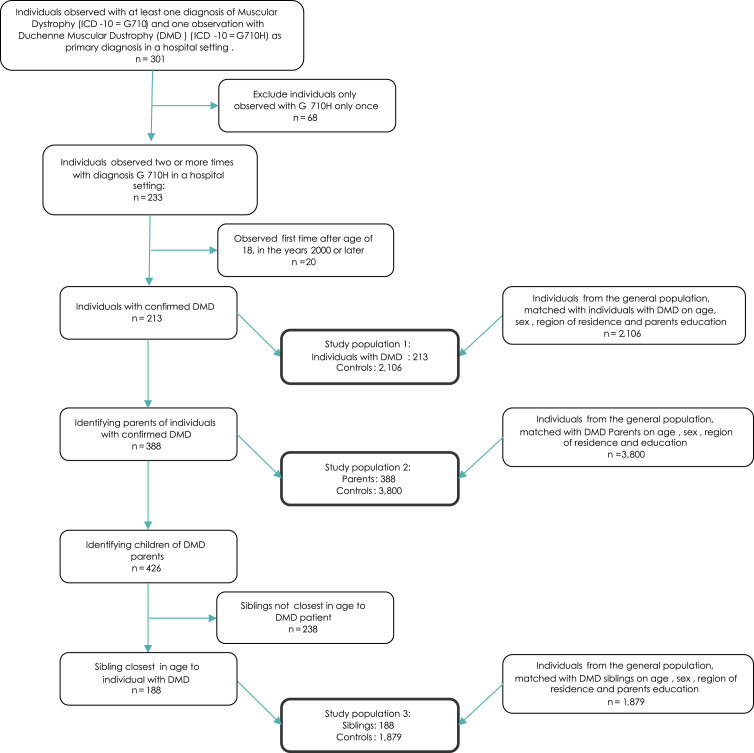
Flowchart of inclusion/exclusion of study population.

During the study period, 301 individuals were observed with the G71.0H diagnosis in the NPR. Of these, 68 individuals were observed only once and were therefore excluded. Furthermore, among those individuals observed with G71.0H more than twice, 20 were aged 18 or older at first observation and these individuals were excluded, as a DMD diagnosis was deemed unlikely to be individuals with DMD. Hence, the DMD study population consisted of 213 individuals with DMD. For these individuals, 388 parents were identified. In total, these parents had 426 children. When only including the sibling closest in age to the individual with DMD following the criteria made in the study, 188 siblings were included in the analysis. In the sibling population, 26 individuals had DMD themselves –i.e., 26 individuals were included both as a individual with DMD, and a DMD sibling. This was accounted for by adjusting for whether the sibling had DMD in all analyses of the sibling population. Figure 1 presents a flowchart of how the study population was identified.

Baseline statistics are presented in Table 1. The median age at diagnosis was 6 years, with inter quartile range (IQR) 4–10 years old. Siblings were slightly older (median: 7, IQR: 4–12) while the median age of parents was 37 years old at DMD diagnosis (IQR: 34–42). Fewer than five individuals with DMD were female, while there was a balanced sex distribution among parents and siblings.

**Table 1:** Summary statistics at baseline^1^

	Individuals with DMD		Parents		Sibling	
	Individuals with DMD	Controls	Parents	Controls	Siblings	Controls
N	213	2106	388	3800	188	1879
Age	6 (4, 10)	6 (4, 10)	37 (33, 42)	37 (34, 42)	7 (4, 12)	7 (4, 12)
Sex^2^
Female	< 5	< 5	198 (51%)	1,937 (51%)	86 (46%)	860 (46%)
Male	> 5	> 5	190 (49%)	1,863 (49%)	102 (54%)	1,019 (54%)
Region
Capital Region	47 (22%)	459 (22%)	84 (22%)	813 (21%)	42 (22%)	420 (22%)
Central Region	51 (24%)	505 (24%)	98 (25%)	955 (25%)	44 (23%)	439 (23%)
Region North	29 (14%)	290 (14%)	50 (13%)	488 (13%)	27 (14%)	270 (14%)
Region Zealand	27 (13%)	270 (13%)	49 (13%)	482 (13%)	22 (12%)	220 (12%)
Region South	59 (28%)	582 (28%)	107 (28%)	1,062 (28%)	53 (28%)	530 (28%)
Has DMD	213 (100%)				26 (14%)

Notes: ^1^Summary statistics at time of diagnosis for individuals with DMD, parents, siblings closest in age and their respective control groups. Some of the individuals with DMD (< 5) received gender affirming treatment, and legally changed their gender. In the study we applied their birth sex throughout the study period. ^2^Due to the rules of discretion using data from Danish registers, i.e., when an output is below 5, which has to secure that a person/patient cannot be identified in a presentation of result, females have to be reported as < 5 in the table. As gender is a dichotomous variable the exact number of males in the patient and control groups can neither be reported other than > 5, because the exact number of females then could be calculated from this.

**Fig. 2:**
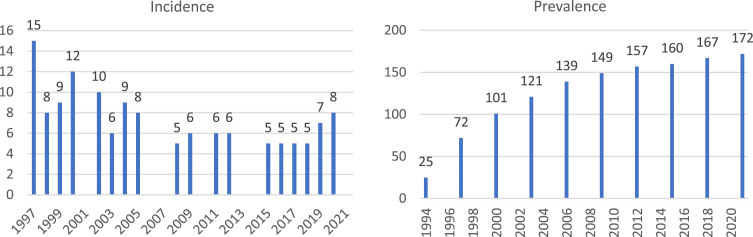
Incidence and prevalence of DMD, number of individuals with DMD per year. Note: Incidence in years 2001, 2006, 2007, 2010, 2013 and 2014 is not provided due to the incidence being lower than five. Prevalence is provided every third year due to data privacy regulations. Incidence in the first few years is likely overestimated due to prevalent individuals from before 1997 being observed with relevant ICD-10 code for the first time in the registers.

The median incidence of DMD in Denmark during the years 1997–2021 was 6.5. The years 1994–1996 were considered a wash-out period. The prevalent DMD population in 2021 was 172. As displayed in Fig. 2, the prevalence is increasing, while the incidence has a stationary trend, presumably due to current individuals with DMD longer survival.

### Clinical outcomes

Individuals with DMD had 23.3 times higher mortality hazard compared to their matched controls. In total, 46 individuals with DMD died during the study period. Mean age at death was DMD 26.8 years (IQR: 19–34). There was no difference in risk of death for parents.

Table 2 presents the most frequent cause of contact with hospital care in the three study populations. The two most frequent causes of contacts were related to ruling out diseases. Furthermore, 73% of the study population with DMD was observed with respiratory failure, 67% observed with dependency on assistive machines such as respirator or wheelchair, and 54% had a scoliosis diagnosis.

**Table 2:** Most frequent diagnosis codes observed for individuals with DMD, siblings and parents stratified by age groups

	Individuals with DMD (N = 213)					Parents (N = 388)					Siblings (N = 188)				
	Diagnosis and ICD-10 code					Diagnosis and ICD-10 code					Diagnosis and ICD-10 code				
	Contact for medical observation for suspected diseases and conditions ruled out	Contact for other special examination without complaint, suspected or reported diagnosis	Respiratory failure, not elsewhere classified	Dependence on enabling machines and devices, not elsewhere classified	Scoliosis	Contact for other special examination without complaint, suspected or reported diagnosis	Contact for medical observation for suspected diseases and conditions ruled out	Persons contacting health services in other circumstances	Contact for follow-up examination after completed treatment for conditions other than malignant neoplasm	Outcome of delivery	Contact for other special examination without complaint, suspected or reported diagnosis	Contact for medical observation for suspected diseases and conditions ruled out	Open wound of head	Dislocation and sprain of joints and ligaments at ankle, foot and toe level	Superficial injury of wrist, hand and fingers
Age group	DZ03	DZ01	DJ96	DZ99	DM41	DZ01	DZ03	DZ76	DZ09	DZ37	DZ01	DZ03	DS01	DS93	DS60
0 to 7	74%	41%	8%	< 5	< 5	–	–	–	–	–	11%	2%	15%	< 5	< 5
8 to 11	16%	26%	16%	11%	10%	–	–	–	–	–	< 5	< 5	< 5	6%	6%
12 to 17	22%	23%	50%	39%	50%	–	–	–	–	–	18%	6%	3%	7%	9%
18 plus	22%	32%	33%	42%	11%	88%	73%	27%	26%	23%	23%	22%	5%	9%	7%
Total	**100% **	**90% **	**73% **	**67% **	**54% **	**88% **	**73% **	**27% **	**26% **	**23% **	**39% **	**35% **	**18% **	**17% **	**16% **

Note: Only the first observation with treatment code per individual was used. The total is a percentage of individuals observed with a procedure code out of all individuals in the study group.

For siblings the most frequent contacts were also related to examinations due to suspected disease or to rule out conditions. Other frequent causes of contacts were related to contact to medical services because of wounds or superficial injuries. Likewise, the most frequent diagnosis code for parents was for exa-minations without complaints or specific diagnosis. Parents also had frequent contacts with the healthcare system under other circumstances.

For selected treatments, in individuals with DMD 12 years of age or older, 39% and 59% were diagnosed with scoliosis or heart disease, respectively. 35% of individuals with DMD underwent Achilles tendon surgery before the age of 18, and 86% of individuals with DMD aged 8 years or older had respiratory failure (Supplementary Table 1). First re-gistration of dependency of wheelchair and respirator is presented in Supplementary Table 2.

Lastly, the most frequent surgeries for individuals with DMD were surgery of the spine or Achilles tendon. Furthermore, bronchoscopies and tracheotomies were frequent surgical procedures. First incidence stratified by age for these surgeries are presented in Table 3.

**Table 3:** Most frequent surgeries observed for individuals with DMD by age-groups

	Surgery				
	Bronchoscopies	Tracheotomies	Joint resections, arthroplasties	Transplants	Operations on muscles and tendons
			and arthrodeses in the spine	on columna	in the knee and lower leg
Age group	KUGC	KGBA	KNAG	KNAN	KNHL
0 to 7	–	–	–	–	7% (11/163)
8 to 11	–	–	–	–	21% (34/164)
12 to 17	8% (14/167)	11% (19/167)	49% (82/167)	43% (72/167)	10% (17/167)
18 plus	33% (46/139)	24% (34/139)	–	4% (6/139)	–
Total	**28% **	**25% **	**38% **	**37% **	**29% **

Note: Only the first observation with treatment code per individual with DMD was used. Table cells contain the number of individuals undergoing the procedure divided by the total number of unique individuals observed in the age groups. Not all individuals were observed in all age groups. Some individuals are observed in multiple age groups. The total is a percentage of individuals observed with a procedure code out of the total study population.

Mean age at first respiratory mask treatment was 15.3 years (standard deviation: 6.7 years). 141 individuals with DMD were observed receiving this treatment. Start of in-home mechanical respirator was at mean age 23.2, with 127 individuals with DMD being registered with in-home mechanical ventilation.

### Use and costs of healthcare services

**Fig. 3:**
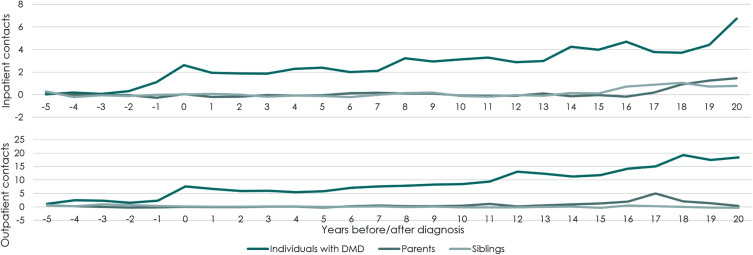
Number of inpatient and outpatient hospital contacts per year attributable to DMD. Note: Inpatient contacts in top graph. Outpatient contacts in bottom graph. Attributable contacts calculated as the difference in contacts between the DMD related group and the respective control populations. Outpatient contacts defined as hospital contacts lasting shorter than 12 hours.

Individuals with DMD exhibited higher use of hospital care in three out of the five years before their DMD diagnosis compared to their controls. Figure 3 presents the extra number of inpatient and outpatient contacts attributable to DMD each year during the study period. After diagnosis, individuals with DMD had between two and seven hospital admissions more compared to their controls each year, and six to 19 additional outpatient contacts. In the 20 years after diagnosis, each individual with DMD had on average 60 additional hospital admissions and 200 additional outpatient contacts compared to their controls. Part of this is potentially due to treatment as 206 out of 213 individuals with DMD have received corticosteroids at least once with the aim to stabilize muscle strength for a period of time. Moreover, individuals with DMD received significantly more of these services in five of the last nine years of the study period. Furthermore, individuals with DMD received significantly more home care intended for personal care in ten years of the study period. The largest difference was found in year 14 after diagnosis, where they received 85 hours more on average than their controls.

**Fig. 4:**
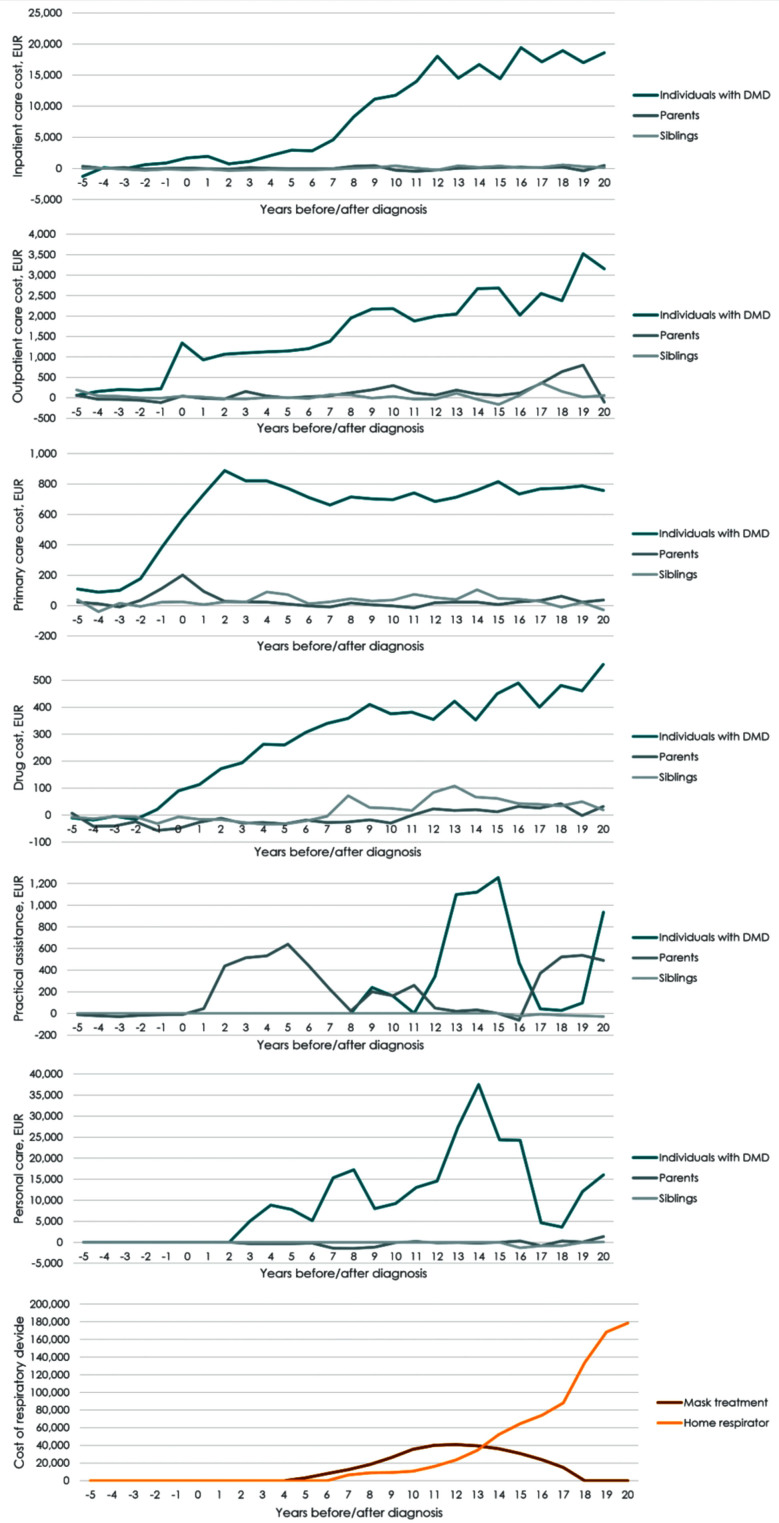
Mean annual attributable costs of healthcare services per individual with DMD by cost category (EUR). Note: Mean total costs are the sum of hospital care, primary care, prescription medication, home care services, non-invasive ventilation and in-home respirator per year. Attributable costs are calculated as the mean costs per individual with DMD minus the mean costs for the controls.

Parents and siblings of individuals with DMD exhibited higher use of healthcare services towards the end of the study period, with higher utilisation of hospital care. Parents had significantly higher use of inpatient or outpatient care in seven out of the last ten years in the study. Siblings had significantly higher use of inpatient care in the last five years of the study. Parents of individuals with DMD received significantly more home care intended for practical assistance in three out of the first six years after diagnosis. For parents, increased costs of primary care services were observed from the year before diagnosis and in the two years following diagnosis.The additional utilisation of hospital care was reflected in the costs of care attributable to DMD. Figure 4 presents the total extra costs of care per individual with DMD, parent, or sibling during the study period, by cost category (Supplementary Tables 3–5 present the numeric value in each period). The total extra costs consisted of hospital care, primary care, prescription drugs, home care, non-invasive ventilation and 24-hour in-home mechanical ventilation. In the 20 years following diagnosis, the total extra direct costs due to DMD per individual summed to EUR 221,500 for inpatient care, EUR 37,600 for outpatient care, EUR 15,000 for primary care, and EUR 6,400 in prescription drug costs and EUR 32,500 in home care costs. If the individual with DMD had a constant annual cost of EUR 125,400 from implementation of non-invasive ventilation until they started in-home mechanical respiration, die or reach end of follow up, the extra costs of non-invasive ventilation accounted for EUR 331,500. If individuals with DMD had a constant annual cost of EUR 211,000 for in-home mecha-nical respirator from start of in-home mechanical respiration until they died or reached end of study, the average extra cost of in-home mechanical respiration was EUR 874,900 per individual with DMD. During the 20 years following DMD diagnosis, the mean total, direct extra cost due to DMD per indivi-dual was EUR 1,524,000.

### Labour market outcomes

Parents of individuals with DMD did not exhibit increased risk of early retirement. Furthermore, it was found that parents of individuals with DMD had lower unemployment towards the end of the study period and spent fewer weeks on long-term sick leave compared to their controls. On the other hand, siblings of individuals with DMD were found to have higher unemployment compared to their controls and higher incidence of long-term sick leave.Despite differences in sick leave and unemployment, no significant differences were found in income for parents and siblings compared to their respective control groups.For individuals with DMD, the labour market participation was low. Thirty one of them (14,5%) were observed with income in at least one year. Sixteen out of the 31 was observed with income in three years or fewer, after turning 18. For the ages 18–30, individuals with DMD had an average annual production loss of EUR 20,200 (CI: 19,700–20,800) compared to the controls.

### Education

Individuals with DMD were less likely to obtain higher education (odds ratio (OR): 0.42, CI: 0.24–0.73) or graduate high school (OR: 0.18, CI: 0.12–0.28). They did also obtain lower grades than their controls in high school (OR: 0.59, CI: 0.40–0.86). Moreover, individuals with DMD received special education to a much higher degree than their controls (38% of individuals with DMD and 8% of controls were observed in special education classes), with individuals with DMD spending 11% of their elementary school time in special education. The extra costs of special education for an individual with DMD were EUR 16,400 (CI: 14,100–18,700) per year. Over the course of 11 school years, the total extra added education cost due to DMD was EUR 176,900.For siblings of individuals with DMD the incidence of special education did not differ significantly from the control group (7% of siblings and 6% of their controls were observed at least once in special education classes. Measure made after excluding siblings with DMD). However, siblings of individuals with DMD did spend more time in special education than their controls. On average, each sibling had extra costs of education of EUR 10,200 during these two years. Furthermore, siblings of indivi-duals with DMD showed a tendency to have lower grades in high school than their controls (*p* = 0.08, adjusted for age, sex, and time). These results should be viewed in the context of a small sample size as grades was only observed for 58 siblings and 625controls.

### Age-related subgroup analysis

**Fig. 5:**
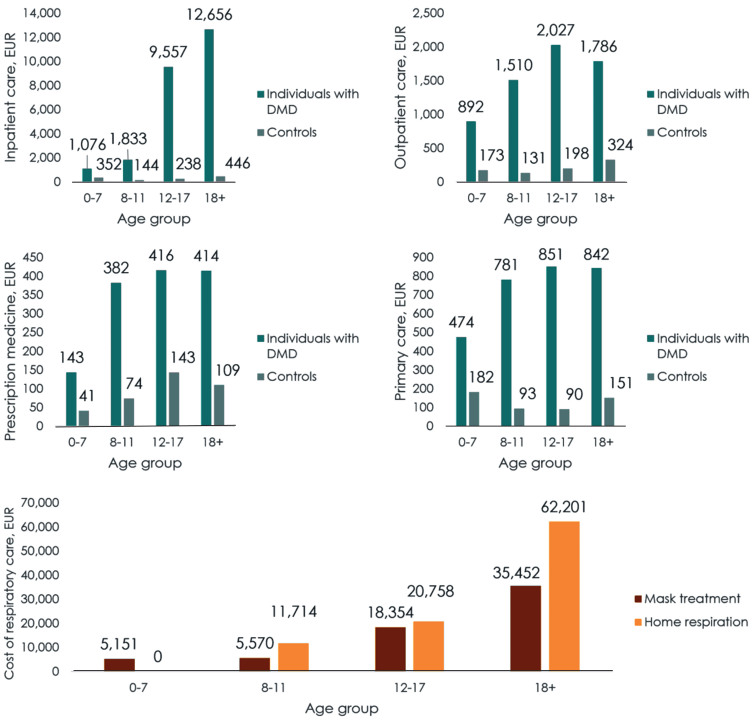
Age-related subgroup analysis of costs per year (EUR). Note: Figures depict costs generated by individuals with DMD and controls, respectively, except for respiratory illnesses. Too few individuals in the control population were identified to be presented as a comparator within data privacy regulations. We found this reasonable, as the control population is relatively young. Most etiologies requiring respiratory treatment are rare, and usually only occur with a higher age at onset. Therefore, the last figure only depicts the costs of respiratory treatments for individuals with DMD.

For inpatient care, the extra costs due to DMD were significantly higher from age 12 onwards. For outpatient hospital care, prescription medicine and primary care services, the extra costs due to DMD were significantly higher in all age groups.Figure 5 presents the total extra costs of care due to DMD each year within each age group. The mean annual extra cost of care due to DMD was EUR 7,800 for individuals with DMD aged 0–7, EUR 21,500 for individuals aged 8–11, EUR 51,700 for individuals aged 12–17, and EUR 113,100 for individuals aged 18 or more.

This means that the extra costs of healthcare services for DMD before an individual with DMD turns 18 years old summed to EUR 452,100, while the extra costs reached EUR 1,922,000 if an individual lives to be 30 years old. When including special education and production loss, the total extra costs of a indivi-dual with DMD who lives to be 30 years old, summed to EUR 2,365,800.

## DISCUSSION

This longitudinal register study makes use of the unique ICD10-diagnosis code for DMD, G71.0H, in the national Danish health registers, implying that the population of individuals with DMD was identified with high certainty. Merging multiple administrative national registers through a person-specific identification number enabled the use of comprehensive Danish register data to depict the disease progression over a 28-year timespan. Using this approach, the study includes a nationwide population with DMD.

The present study found that the use of healthcare services and attributable costs due to DMD significantly increased over time and progression for individuals with DMD compared to the control group. The total direct extra healthcare costs due to DMD were EUR 1,524,000 per average individual with DMD 20 years after diagnosis, and the extra costs of education were found to be EUR 180,900. For an individual with DMD being 30 years old the total extra cost due to DMD was EUR 2,365,800. A result still being conservative as personal assistants as well as production loss from short term sick-leave and premature death were not included. Signs of a burden for parents and siblings –particularly for siblings spen-ding more time on special education, having more sick-leave and unemployment –were also found.

The previously suggested DMD age-related subgroups (age 0–7, 8–11, 12–17, 18+) corresponds well with the plateaus in use of hospital care, presented in Fig. 3. Minor deviations are present, which is to be expected as the previously defined age-related subgroups were set based on a global DMD population. These deviations are likely due to prevalent patients at start of the study period, institutional healthcare setting, lags in register data compared to clinical practice or improved treatments during the study period (e.g., corticosteroids and mechanical ventilation units). There is an argument to be made, however, of an additional end-of-life stage beginning 18 years after diagnosis.

Previous studies have come to similar cost estimates as what was presented here. A 2013 study from the US found the average annual costs of DMD to be USD 50,952 (2013 valuation), corresponding to USD 1,528,560 total costs for a 30 year old individual with DMD [19]. A Portuguese study found that individuals with DMD had a mean annual costs of EUR 49,991 in the non-ambulatory stage, while it was EUR 19,993 during ambulatory stage (both 2019 valuations) [20], corresponding to about EUR 1,179,750 for a 30-year-old individual with DMD. A German study found DMD to generate annual costs of EUR 78,913 (2013 valuation), including indirect and informal care costs [21]. A multinational study of German, Italian, UK and US individuals with DMD found direct cost of illness to be between US 23,920 and USD 54,270, while the societal costs varied from USD 80,120 to USD 120,910 (2012 valuation) [22]. Note, however, that these aforementioned studies were based on survey data of the average patient or cross-sectional data, and authors of these reports recognize that significant costs are absorbed by the household. With the intensive social programs available in Denmark, a significant part of the household costs will likely be transferred as a public expense instead.

The present study showed sporadic signs of the burden of illness for parents and siblings of indivi-duals with DMD. Siblings spend more time on special education than their controls resulting in an extra cost of education during these two years. A tendency for lower school grades were found, although insigni-ficant. Moreover, siblings have higher incidence of long-term sick-leave and higher unemployment than their controls. Granted, these differences are not consistently statistically different throughout the study period. Previous studies of individuals with DMD and their parents reported how parents perceive the challenges faced by individuals with DMD as more severe than the individuals themselves [9]. It is likely that similar worries influence siblings, which in turn result in lower academic performances. It serves as an important reminder that fatigue, worry about the future, vitality and social relations are dimensions of health-related quality of life, which are not captured by the register data.

Furthermore, the differences in costs betweenparents/siblings and their respective controls are evident in some periods, but over the study period the total difference is not of considerable size. This is also the case for early retirement that was not found to be higher for parents of individuals with DMD as for controls. This lack of differences must be seen in context of the institutional setting. First, Danish citizens with DMD have significantly better access to care than citizens with DMD in comparable countries [23]. Second, Denmark has widely available public social security services, which probably lower the impact on informal care givers of severely ill patients [24]. Furthermore, it does at the same time signal the strength and flexibility of parents and siblings of individuals with DMD despite the impact this severe disease makes on their daily living.

### Strengths and weaknesses

This study applied Danish health and administrative registers. These registers are of high completeness and frequently used in scientific research [15]. Utilizing the full national population of individuals with DMD from the registers in the study reduces the risk of inclusion bias related to patient selection. Furthermore, the entire Danish population was available as matches, ensuring suitable control populations for individuals with DMD and their relatives. However, in register-based research, there is always the risk of wrongful inclusion or exclusion of patients. When comparing incidence and prevalence in the years 1997–2001 with a previously published study in Denmark based on journal review [25], the prevalence found in the present study was likely underestimated in the first 10 years of the study period. The potential bias of this resulted in an underestimation of the total costs in the results, as we are more likely to observe the later stages of DMD in individuals diagnosed early in the study period. However, The Danish Rehabilitation Centre for Neuromuscular Diseases, Aarhus, Denmark, that register all individuals with DMD in Denmark estimated a 2021 prevalence of DMD in Denmark to 170 individuals in 2021, compared to the 172 we identified in the present study. Furthermore, some siblings (fewer than five) were observed with hospital-diagnosed muscular dystrophy (ICD-10: G71.0) at least once during the study, but not the DMD-specific ICD-10 code G71.OH. It is not possible to confirm or rule out whether these cases were individuals with DMD or not based on register data. Due to privacy regulation, additional detail cannot be reported on these individuals, as it is only relevant for fewer than five individuals. It should be highlighted that since it is only valid for such few individuals, the influence it could have had on the results is minor. Finally, for eventual divorced parents we were not able to see custody distribution for the parent that the child is not living together with, as the child can only be registered with one address in the CPR register, and i.e., parent. Our best ex-ante assumption though was the divorce rates should not differ significantly between the case and control group and the impact being very limited.

Start of respiratory treatments, either with a mask or full-time in-home mechanical respirator, are subject to discussion. However, the age at incidence in this study coincided well with age at incidence from other studies [25–27]. As evident in the data, there was steep decrease in home care services 17 years after diagnosis, which corresponds with start of respiratory treatment, where the responsibility of home care services is transferred from the municipality to specialized care facilities based in public hospitals. Furthermore, the costs associated with the treatments were conservative, as they omitted the purchase of equipment and administrative costs related to treatment.

The measure of long-term sick leave might not be the most suitable measure to determine work absenteeism in this context. The measure does not display periods of sick leave shorter than four weeks, which might bias the results. It is unfortunate that data on short-term sick leave was not available, why the costs estimated in terms of production lost are conservative. Furthermore, parents are compensated for lost wages or added costs associated with supporting their DMD children. These costs could not be identified in the available registers but would have added to the overall total costs of DMD had they beenincluded.

Variation in use of corticosteroids may have been influential on the number and timing of contacts as majority of the individuals with DMD had received corticosteroids at least once. Variations due to corticosteroids treatment was however considered out of scope for the study.

The use of corticosteroids also highlights an important point related to treatment regimens. During the last 28 years, guidelines on how to treat DMD have changed. Fewer individuals with DMD undergo surgery in the more recent periods, compared to the start of the study. Therefore, there might be cohort effects which could have influenced theresults.

Analysis of use of personal assistants and aid in relation to physical handicaps was planned, but the data were too sparse to provide reliable results. The reporting to the Danish register of disability recipients is subject to municipal practices, which turned out to be too inconsistent to be applied in this setting. As this was then omitted from the analysis just made the costs results even more conservative.

Finally, the productivity loss for individuals with DMD is a conservative estimate. Individuals in the control group were likely to pursue education and therefore delay a steady income during the ages 18–30 years, while potential production lost due to premature death of individuals with DMD were omitted from the analysis.

In conclusion, the study showed that the use of healthcare services for individuals with DMD were significantly increasing over time as disease progresses. Similarly, the costs of care due to DMD increased as the disease progressed and, lead to high extra costs of the DMD disease compared to matched controls without DMD. The total extra cost due to DMD sums up to EUR 1,922,000, or EUR 2,365,800 including special education and production loss, if an individual with DMD lives to be 30 years old. This is a conservative estimate of the total extra cost due to DMD as some data for some costs, e.g., use of personal assistants and aid and parents short term sick leave, were not available, as well as the production loss due to premature death of individuals with DMD was omitted. The burden experienced by siblings was not evident in the register data. There is, however, indications of reduced educational performance as well as higher unemployment and higher incidence of long-term sick leave for siblings compared to their controls. Compared to matched controls without DMD the burden of DMD was therefore high. In the register data, the societal burden of disease is primarily observed for the individual with DMD, but evidence of burden for parents and siblings of indivi-duals with DMD was also found. Future survey studies should strive to include dimensions of health-related quality of life for the informal caregivers as well as investigate the short-term productivity loss and absenteeism of parents, not captured in the register data.

## Supplementary Material

Supplementary Material

## DATASETS

The data that support the findings of this study are available from Statistics Denmark’s Research Service, but restrictions apply to the availability of these data, which were used under license for the current study, and so are not publicly available.

## References

[1] Mendell JR , Shilling C , Leslie ND , Flanigan KM , al-Dahhak R , Gastier-Foster J , et al. Evidence-based path to newborn screening for duchenne muscular dystrophy. Ann Neurol 2012;71(3):304–13. 22451200 10.1002/ana.23528

[2] Moat SJ , Bradley DM , Salmon R , Clarke A , Hartley L . Newborn bloodspot screening for Duchenne Muscular Dystrophy: 21 years experience in Wales (UK). Eur J Hum Genet 2013;21(10):1049–53. 23340516 10.1038/ejhg.2012.301 PMC3778339

[3] Duchenne muscular dystrophy: about the disease[Internet]. 2021 [cited 2022 Nov 9]. Available from:https://rarediseases.info.nih.gov/diseases/6291/duchennemuscular-dystrophy.

[4] Duchennes muskeldystrofi (DMD) [Internet]. RCFM.[cited 2022 Nov 9]. Available from:https://rcfm.dk/diagnose/duchenns-muskeldystrofi-dmd/.

[5] Uzark K , King E , Cripe L , Spicer R , Sage J , Kinnett K , et al. Health-Related Quality of Life in Children and Adolescents With Duchenne Muscular Dystrophy. Pediatrics 2012;130(6):e1559–66. 23129083 10.1542/peds.2012-0858

[6] Landfeldt E , Thompson R , Sejersen T , McMillan HJ , Kirschner J , Lochmüller H . Life expectancy at birth in Duchenne muscular dystrophy: a systematic review and meta-analysis. Eur J Epidemiol 2020;35(7):643–53. 32107739 10.1007/s10654-020-00613-8 PMC7387367

[7] Orphanet: Duchenne muscular dystrophy [Internet].[cited 2022 Nov 9]. Available from:https://www.orpha.net/consor/cgi-bin/Disease_Search.php?lng=EN&data_id=13913&Disease_Disease_Search_diseaseGro.

[8] Broomfield J , Hill M , Guglieri M , Crowther M , Abrams K . Life Expectancy in Duchenne Muscular Dystrophy: Reproduced Individual Patient Data Meta-analysis. Neurology 2021;97(23):e2304–14. 34645707 10.1212/WNL.0000000000012910 PMC8665435

[9] Handberg C , Werlauff U , Hensuremath øjberg AL . Perspectives on Everyday Life Challenges of Danish Young People With Duchenne Muscular Dystrophy (DMD) on Corticosteroids. Global Qualitative Nursing Research 2022;9:233339362210948. 10.1177/23333936221094858 PMC9052227 35493771

[10] Duan D , Goemans N , Takeda S , Mercuri E , Aartsma-Rus A . Duchenne muscular dystrophy. Nature Reviews Disease Primers 2021;7(1):13. 10.1038/s41572-021-00248-3 PMC10557455 33602943

[11] Ryder S , Leadley RM , Armstrong N , Westwood M , de Kock S , Butt T et al. The burden, epidemiology, costs and treatment for Duchenne muscular dystrophy: an evidence review. Orphanet J Rare Dis 2017;12(1):79. 28446219 10.1186/s13023-017-0631-3 PMC5405509

[12] Landfeldt E , Edström J , Buccella F , Kirschner J , Lochmüller H . Duchenne muscular dystrophy and caregiver burden: a systematic review. Dev Med Child Neurol 2018;60(10):987–96. 29904912 10.1111/dmcn.13934

[13] Lynge E , Sandegaard JL , Rebolj M . The Danish National Patient Register. Scand J Public Health 2011;39(7):30–3. 21775347 10.1177/1403494811401482

[14] Hjollund NH , Larsen FB , Andersen JH . Register-based follow-up of social benefits and other transfer payments: Accuracy and degree of completeness in a Danish interdepartmental administrative database compared with a population-based survey. Scand J Public Health 2007;35(5):497–502. 17852980 10.1080/14034940701271882

[15] Thor Petersen C , Jensen KJ , Rosenzweig M , von Osmanski BI , Ankarfeldt MZ , Petersen J . Mapping Outcomes and Registries Used in Current Danish Pharmacoepidemiological Research. CLEP 2022;14:521–42. 10.2147/CLEP.S341480 PMC9056023 35502197

[16] PRIS112: Consumer price index (2015=100) by main figures [Internet]. Statistics Denmark; 2023 Jan. Available from:https://www.statbank.dk/statbank5a/selectvarval/de-fine.asp?PLanguage=1&subword=tabsel&MainTable=PR-IS112&PXSId=194807&tablestyle=&ST=SD&buttons=0.

[17] Så meget koster en skoleelev: kommunernes enhedsudgifter påskoleområdet 2009/2010-2012/2013.2015.

[18] Medicinsk teknologivurdering af respiratorbehandling i eget hjem. Aarhus: DEFACTUM, Region Midtjylland;2017.

[19] Larkindale J , Yang W , Hogan PF , Simon CJ , Zhang Y , Jain A et al. Cost of illness for neuromuscular diseases in the United States: Cost of Illness for NMD. Muscle Nerve 2014;49(3):431–8. 23836444 10.1002/mus.23942

[20] Labisa P , Andreozzi V , Mota M , Monteiro S , Alves R , Almeida J et al. Cost of Illness in Patients with Duchenne Muscular Dystrophy in Portugal: The COIDUCH Study. PharmacoEconomics Open 2022;6(2):211–8. 34604937 10.1007/s41669-021-00303-5 PMC8864047

[21] Schreiber-Katz O , Klug C , Thiele S , Schorling E , Zowe J , Reilich P et al. Comparative cost of illness analysis and assessment of health care burden of Duchenne and Becker muscular dystrophies in Germany. Orphanet J Rare Dis 2014;9(1):210. 25519771 10.1186/s13023-014-0210-9 PMC4302713

[22] Landfeldt E , Lindgren P , Bell CF , Schmitt C , Guglieri M , Straub V et al. The burden of Duchenne muscular dystrophy: An international, cross-sectional study. Neurology 2014;83(6):529–36. 24991029 10.1212/WNL.0000000000000669 PMC4141999

[23] Rodger S , Woods KL , Bladen CL , Stringer A , Vry J , Gramsch K et al. Adult care for Duchenne muscular dystrophy in the UK. J Neurol 2015;262(3):629–41. 25536903 10.1007/s00415-014-7585-3 PMC4363521

[24] Fadlon I , Nielsen TH . Family Labor Supply Responses to Severe Health Shocks: Evidence from Danish Administrative Records. American Economic Journal: Applied Economics 2021;13(3):1–30.

[25] Jeppesen J , Green A , Steffensen BF , Rahbek J . The Duchenne muscular dystrophy population in Denmark, 1977-2001: prevalence, incidence and survival in relation to the introduction of ventilator use. Neuromuscular Disorders 2003;13(10):804–12. 14678803 10.1016/s0960-8966(03)00162-7

[26] MacKintosh EW , Chen ML , Benditt JO . Lifetime Care of Duchenne Muscular Dystrophy. Sleep Medicine Clinics 2020;15(4):485–95. 33131659 10.1016/j.jsmc.2020.08.011 PMC7534837

[27] Dreyer PS , Steffensen BF , Pedersen BD . Life with home mechanical ventilation for young men with Duchenne muscular dystrophy. Journal of Advanced Nursing 2010;66(4):753–62. 20423363 10.1111/j.1365-2648.2009.05233.x

