# Supplementary material for: Burden of Disease of Duchenne Muscular Dystrophy in Denmark – A National Register-Based Study of Individuals with Duchenne Muscular Dystrophy and their Closest Relatives
Source: J Neuromuscul Dis. 2024 Mar 5;11(2):443–57. doi: 10.3233/JND-230133 (PMC10977402; doi:10.3233/JND-230133)
Supplement: Supplementary Material [file jnd-11-jnd230133-s001.docx]

**Supplementary Table 1: Criteria for identification of selected treatments; scoliosis surgery, Achilles surgery, respiratory illnesses, and heart disease**

|  | **Scoliosis** | | **Achilles** | **Respiratory illnesses** | | **Heart disease** |
| --- | --- | --- | --- | --- | --- | --- |
|  | **ICD-10** | **Procedure** | **Procedure** | **ICD-10** | **Procedure** | **ICD-10** |
|  | M41 (Scoliosis) | KNAG7 (Posterior spondylodosis with fixation) | KNHL (Operations on muscles and tendons in the ankle and foot) | Z991 (Dependence on respirator) | KGBA (Tracheotomies, removal of pathological tissue and operations for lesions) | I110 (Hypertensive heart disease with heart failure) |
|  | Q76  (Congenital malformations of spina and bony thorax) | KNAN0 (Autotransplantation of bone on columna) |  | Z930 (Tracheostomy status) | KGBB (Tracheostomies and associated operations) | I130 (Hypertensive heart and chronic kidney disease with heart failure and stage 1 through stage 4 chronic kidney disease, or unspecified chronic kidney disease) |
|  |  | KNAN1 (Allotransplantation of bone on the spine) |  | Any starting with J (Disease of respiratory organs) | BGFC (Lung therapy and other respiratory treatment) | I320 (Pericarditis in diseases classified elsewhere) |
|  |  | KNAT2 (Posterior traction and correction with internal fixation in the columna) |  |  |  | I420 (Dilated cardiomyopathy) |
|  |  | KNAG4 (Anterior spondylodesis with internal fixation) |  |  |  | I426 (Alcoholic cardiomyopathy) |
|  |  | KNAG5 (Anterior spondylodosis with external fixation) |  |  |  | I427 (Cardiomyopathy due to drug and external agent) |
|  |  | KNAK1 (Resection or excision of vertebra) |  |  |  | I429 (Cardiomyopathy, unspecified) |
|  |  |  |  |  |  | I500 (Heart failure) |
|  |  |  |  |  |  | I509 (Heart failure, unspecified) |
| Age group |  | |  |  | |  |
| 0 to 7 | <5 | | 8 (5%) | <5 | | <5 |
| 8 to 11 | <5 | | 32 (20%) | 7 (4%) | | <5 |
| 12 to 17 | 58 (35%) | | 18 (11%) | 67 (40%) | | 22 (13%) |
| 18 Plus | 6 (4%) | | <5 | 57 (41%) | | 64 (46%) |

Note: For groups including both ICD-10 code and procedure code, the two had to be observed in the same hospital contact to be included as treatment. Lower part of the table present incidence by age group.

**Supplementary Table 2: Age specific prevalence of dependency of wheelchair and dependency of respirator**

| Age group | Unique observations in age group | Cumulative events, wheelchair | New events wheelchair | Observed as pct of potentially observed, wheelchair | Cumulative observed, respirator | New events in group, respirator | Observed as pct. potentially observed with respirator |
| --- | --- | --- | --- | --- | --- | --- | --- |
| 0 to 7 | 163 | <5 | <5 | <5 | <5 | <5 | <5 |
| 8 to 11 | 164 | 28 | <5 | 0.17 | <5 | <5 | <5 |
| 12 to 17 | 167 | 81 | 53 | 0.49 | 36 | 34 | 0.22 |
| 18 plus | 139 | 122 | 41 | 0.88 | 108 | 72 | 0.78 |

Note: Not all individuals are observed in all age groups. As the dependency is chronic, an individual observed as dependent on wheelchair or respirator in the age group ’12 to 17’ will also be dependent on wheelchair or respirator, if observed in the age group ’18 plus’. Hence, a cumulative percentage of dependence is provided.

**Supplementary Table 3: Extra costs attributable to DMD for individuals with DMD by period, for cost categories: inpatient care, outpatient care, primary care, drug costs, home care, mask respiration, in home mechanical respirator. Column to the right is the sum of all cost categories, i.e., total extra costs due to DMD per period (EUR)**

| Individuals with DMD | | | | | | | | | |  |
| --- | --- | --- | --- | --- | --- | --- | --- | --- | --- | --- |
| Periode | Inpatient contacts | Outpatient contact | Primary caare | Drug costs | Personal care | Practical aid | non-invasive ventilator | In home mechanical ventilator | **Total costs** | |
| -5 | -1,266 | 59 | 110 | -11 | 0 | 0 | - | - | -1,108 | |
| -4 | 115 | 156 | 89 | -18 | 0 | 0 | - | - | 343 | |
| -3 | -88 | 200 | 101 | -5 | 0 | 0 | - | - | 208 | |
| -2 | 584 | 190 | 178 | -16 | 0 | 0 | - | - | 936 | |
| -1 | 860 | 218 | 382 | 20 | 0 | 0 | - | - | 1,480 | |
| 0 | 1,657 | 1,343 | 569 | 89 | 0 | 0 | - | - | 3,659 | |
| 1 | 1,918 | 925 | 731 | 113 | 0 | 0 | - | - | 3,687 | |
| 2 | 745 | 1,067 | 888 | 172 | 0 | 0 | - | - | 2,871 | |
| 3 | 1,110 | 1,097 | 820 | 194 | 5,026 | 0 | - | - | 8,247 | |
| 4 | 2,067 | 1,126 | 820 | 262 | 8,854 | 0 | - | - | 13,130 | |
| 5 | 2,932 | 1,142 | 771 | 260 | 7,833 | 0 | 3,101 | - | 16,040 | |
| 6 | 2,839 | 1,203 | 712 | 307 | 5,167 | 0 | 7,923 | - | 18,151 | |
| 7 | 4,598 | 1,379 | 662 | 340 | 15,304 | 0 | 12,466 | 6,351 | 41,100 | |
| 8 | 8,300 | 1,951 | 715 | 358 | 17,237 | 0 | 18,234 | 8,811 | 55,606 | |
| 9 | 11,148 | 2,171 | 702 | 410 | 8,004 | 239 | 26,007 | 9,283 | 57,965 | |
| 10 | 11,753 | 2,177 | 698 | 376 | 9,194 | 160 | 35,637 | 10,551 | 70,545 | |
| 11 | 14,002 | 1,874 | 741 | 381 | 13,063 | 0 | 40,068 | 16,083 | 86,213 | |
| 12 | 18,047 | 1,997 | 684 | 354 | 14,556 | 340 | 40,770 | 23,574 | 100,323 | |
| 13 | 14,489 | 2,048 | 713 | 422 | 27,431 | 1,099 | 39,492 | 34,357 | 120,051 | |
| 14 | 16,692 | 2,672 | 758 | 352 | 37,503 | 1,120 | 36,279 | 52,146 | 147,521 | |
| 15 | 14,398 | 2,685 | 816 | 450 | 24,381 | 1,255 | 30,744 | 64,786 | 139,515 | |
| 16 | 19,398 | 2,019 | 735 | 489 | 24,227 | 468 | 23,749 | 74,057 | 145,141 | |
| 17 | 17,113 | 2,550 | 767 | 400 | 4,603 | 43 | 14,847 | 88,150 | 128,473 | |
| 18 | 18,935 | 2,374 | 773 | 480 | 3,626 | 27 | - | 133,762 | 159,977 | |
| 19 | 16,980 | 3,520 | 786 | 460 | 12,101 | 95 | - | 168,394 | 202,337 | |
| 20 | 18,575 | 3,151 | 757 | 559 | 16,054 | 936 | - | 178,600 | 218,631 | |

Note: Costs in Euros, 2021 valuation. Attributable costs calculated as mean costs of individuals with DMD minus mean costs of matched controls.

**Supplementary Table 4: Extra costs attributable to DMD for *parents* by period, for cost categories: inpatient care, outpatient care, primary care, drug costs, home care. Column to the right is the sum of all cost categories, i.e., total extra costs due to DMD per period (EUR)**

| Parents | | | | | | | |
| --- | --- | --- | --- | --- | --- | --- | --- |
| Periode | Inpatient contacts | Outpatient contact | Primary caare | Drug costs | Personal care | Practical aid | **Total costs** |
| -5 | 328 | 61 | 23 | 7 | 0 | -13 | 406 |
| -4 | 8 | -36 | 13 | -42 | 0 | -22 | -79 |
| -3 | 145 | -40 | -8 | -41 | 0 | -29 | 27 |
| -2 | -131 | -64 | 35 | -25 | 0 | -17 | -203 |
| -1 | -18 | -117 | 113 | -57 | 0 | -13 | -93 |
| 0 | 21 | 41 | 201 | -50 | 0 | -11 | 202 |
| 1 | -33 | -16 | 94 | -27 | 0 | 45 | 64 |
| 2 | -144 | -29 | 28 | -12 | 0 | 437 | 279 |
| 3 | 116 | 152 | 25 | -30 | -314 | 514 | 464 |
| 4 | -6 | 42 | 24 | -28 | -308 | 531 | 256 |
| 5 | -40 | -4 | 10 | -32 | -367 | 639 | 206 |
| 6 | -47 | 25 | -2 | -19 | -225 | 441 | 173 |
| 7 | -64 | 50 | -8 | -27 | -1,411 | 223 | -1,237 |
| 8 | 359 | 120 | 17 | -25 | -1,464 | 23 | -971 |
| 9 | 459 | 194 | 5 | -18 | -1,147 | 201 | -306 |
| 10 | -311 | 297 | -1 | -30 | -97 | 162 | 19 |
| 11 | -482 | 121 | -15 | -1 | 150 | 261 | 35 |
| 12 | -245 | 62 | 19 | 23 | -149 | 48 | -242 |
| 13 | 44 | 185 | 24 | 17 | -112 | 20 | 177 |
| 14 | 96 | 91 | 22 | 20 | -242 | 33 | 20 |
| 15 | 147 | 59 | 8 | 12 | -4 | 0 | 222 |
| 16 | 201 | 113 | 24 | 32 | 294 | -63 | 602 |
| 17 | 132 | 353 | 33 | 25 | -817 | 372 | 100 |
| 18 | 197 | 641 | 61 | 42 | 288 | 523 | 1,752 |
| 19 | -385 | 796 | 24 | -2 | 71 | 537 | 1,040 |
| 20 | 455 | -108 | 37 | 32 | 1,355 | 490 | 2,261 |

Note: Costs in Euros, 2021 valuation. Attributable costs calculated as mean costs of DMD parents minus mean costs of matched controls.

**Supplementary Table 5: Extra costs attributable to DMD for *siblings* by period, for cost categories: inpatient care, outpatient care, primary care, drug costs, home care. Column to the right is the sum of all cost categories, i.e., total extra costs due to DMD per period (EUR)**

| Siblings | | | | | | | |
| --- | --- | --- | --- | --- | --- | --- | --- |
| Periode | Inpatient contacts | Outpatient contact | Primary caare | Drug costs | Personal care | Practical aid | **Total costs** |
| -5 | -19 | 193 | 39 | -8 | 0 | 0 | 205 |
| -4 | 30 | 50 | -40 | -14 | 0 | 0 | 26 |
| -3 | -141 | 39 | 16 | -6 | 0 | 0 | -92 |
| -2 | -309 | 0 | -5 | -6 | 0 | 0 | -321 |
| -1 | -141 | -7 | 23 | -32 | 0 | 0 | -158 |
| 0 | -201 | 40 | 25 | -7 | 0 | 0 | -143 |
| 1 | -135 | 19 | 7 | -16 | 0 | 0 | -126 |
| 2 | -320 | -19 | 25 | -17 | 0 | 0 | -331 |
| 3 | -262 | -27 | 23 | -27 | 0 | 0 | -293 |
| 4 | -159 | 2 | 90 | -34 | 0 | 0 | -101 |
| 5 | -239 | 1 | 72 | -33 | 0 | 0 | -199 |
| 6 | -233 | -18 | 12 | -22 | 0 | 0 | -261 |
| 7 | -137 | 79 | 24 | -5 | 0 | 0 | -39 |
| 8 | 50 | 66 | 45 | 71 | 0 | 0 | 233 |
| 9 | 141 | -11 | 30 | 27 | 0 | 0 | 187 |
| 10 | 413 | 31 | 38 | 24 | 0 | 0 | 506 |
| 11 | 38 | -35 | 74 | 17 | 0 | 0 | 94 |
| 12 | -243 | -26 | 52 | 83 | 0 | 0 | -133 |
| 13 | 411 | 106 | 41 | 108 | 0 | 0 | 665 |
| 14 | 166 | -42 | 104 | 67 | 0 | 0 | 294 |
| 15 | 369 | -163 | 47 | 61 | 0 | 0 | 315 |
| 16 | 67 | 66 | 42 | 43 | -1,349 | -25 | -1,157 |
| 17 | 155 | 365 | 29 | 39 | -868 | -7 | -287 |
| 18 | 587 | 155 | -11 | 33 | -876 | -17 | -128 |
| 19 | 275 | 14 | 21 | 50 | 0 | -22 | 338 |
| 20 | 113 | 60 | -29 | 19 | 59 | -28 | 194 |

Note: Costs in Euros, 2021 valuation. Attributable costs calculated as mean costs of DMD siblings minus mean costs of matched controls.
